# Preclinical emergence of vandetanib as a potent antitumour agent in mesothelioma: molecular mechanisms underlying its synergistic interaction with pemetrexed and carboplatin

**DOI:** 10.1038/bjc.2011.400

**Published:** 2011-10-04

**Authors:** E Giovannetti, P A Zucali, Y G Assaraf, L G Leon, K Smid, C Alecci, F Giancola, A Destro, L Gianoncelli, E Lorenzi, M Roncalli, A Santoro, G J Peters

**Affiliations:** 1 Department of Medical Oncology, VU University Medical Center, De Boelelaan 1118, 1081 HV Amsterdam, The Netherlands; 2 Department of Medical Oncology and Hematology, Istituto Clinico Humanitas, Via Manzoni 56, 20089 Rozzano, Italy; 3 Department of Biology, The Fred Wyszkowski Cancer Research Laboratory, Technion-Israel Institute of Technology, Haifa 32000, Israel; 4 Department of Human Pathology, Istituto Clinico Humanitas IRCCS, Via Manzoni 56, 20089 Rozzano, Italy

**Keywords:** mesothelioma, targeted agents, EGFR pathway, thymidylate synthase, apoptosis, DNA repair genes

## Abstract

**Background:**

Although pemetrexed, a potent thymidylate synthase (TS) inhibitor, enhances the cytoytoxic effect of platinum compounds against malignant pleural mesothelioma (MPM), novel combinations with effective targeted therapies are warranted. To this end, the current study evaluates new targeted agents and their pharmacological interaction with carboplatin–pemetrexed in human MPM cell lines.

**Methods:**

We treated H2052, H2452, H28 and MSTO-211H cells with carboplatin, pemetrexed and targeted compounds (gefitinib, erlotinib, sorafenib, vandetanib, enzastaurin and ZM447439) and evaluated the modulation of pivotal pathways in drug activity and cancer cell proliferation.

**Results:**

Vandetanib emerged as the compound with the most potent cytotoxic activity, which interacted synergistically with carboplatin and pemetrexed. Drug combinations blocked Akt phosphorylation and increased apoptosis. Vandetanib significantly downregulated epidermal growth factor receptor (EGFR)/Erk/Akt phosphorylation as well as *E2F-1* mRNA and TS mRNA/protein levels. Moreover, pemetrexed decreased Akt phosphorylation and expression of DNA repair genes. Finally, most MPM samples displayed detectable levels of EGFR and TS, the variability of which could be used for patients’ stratification in future trials with vandetanib–pemetrexed–carboplatin combination.

**Conclusion:**

Vandetanib markedly enhances pemetrexed–carboplatin activity against human MPM cells. Induction of apoptosis, modulation of EGFR/Akt/Erk phosphorylation and expression of key determinants for pemetrexed and carboplatin activity contribute to this synergistic interaction, and, together with the expression of these determinants in MPM samples, warrant further clinical investigation.

Malignant pleural mesothelioma (MPM) is a devastating disease arising from mesothelial cells that line the thoracic cavity. The incidence of MPM is increasing throughout the world, and it is expected to peak around 2015–2020 in Western Europe, as a result of widespread exposure to its main aetiological factor, asbestos (Hodgson *et al*, 2005). There are three distinct histologic subtypes of MPM, based on the cell microscopic appearance. The epithelial cell type is the most common, comprising 50–70% of all MPM; it generally responds the best to treatment, and offers the best prognosis. By contrast, sarcomatoid mesothelioma represents 7–20% of MPM cases and is the most aggressive subtype. The biphasic or mixed cell type, as the name implies, is a combination of elements of both the epithelial and sarcomatoid subtypes and its prognosis is intermediate. However, current MPM treatment options do not vary between cell types. Most patients are not amenable to radical surgery, and the primary goals of treatment in this setting are prolongation of survival and palliation. The combination of cisplatin and pemetrexed has recently become the standard first-line treatment of MPM. This regimen significantly improved the response rate (RR; 41.3%), time-to-progression (TTP; 5.7 months), overall survival (OS; 12.1 months) and quality of life, when compared with cisplatin monotherapy (Vogelzang *et al*, 2003). For patients who are unfit to receive cisplatin-based chemotherapy, pemetrexed alone or in combination with carboplatin has been proposed as an alternative treatment. In our phase II study, the pemetrexed–carboplatin combination was well tolerated and active in 102 MPM patients, showing a disease control rate (65%), TTP (6.5 months) and OS (12.7 months) similar to the results achieved with the pemetrexed–cisplatin regimen (Ceresoli *et al*, 2006). However, given the dismal prognosis primarily due to chemoresistance of MPM, the evaluation of new therapeutic agents is warranted. A number of molecular alterations occurring in MPM have been reported, providing deeper insights into its biology and leading to the identification of novel druggable targets (Zucali *et al*, 2011a, 2011b). Therefore, several novel agents have been, or are currently being evaluated, including drugs targeted against the epidermal growth factor receptor (EGFR), vascular endothelial growth factor (VEGF) and different kinases involved in cancer proliferation (Kindler, 2008).

Treatment of MPM patients with the EGFR-tyrosine kinase inhibitors (TKIs) gefitinib and erlotinib failed to improve response/survival (Govindan *et al*, 2005; Garland *et al*, 2007). Similarly, phase II studies of the antiangiogenic agents SU5416, vatalanib, thalidomide and sorafenib demonstrated only modest activity as monotherapy, hence being comparable to other single agents in this disease (Kindler, 2008). The lack of the expected positive outcome may have been caused by the lack of patient selection, due to the absence of predictive markers for response to targeted agents in MPM, or by the lack of the right chemotherapy cornerstone for the addition of these targeted therapies. More recently, a randomised phase II trial evaluating the addition of the anti-VEGF antibody bevacizumab to gemcitabine+cisplatin reported a slight increase in progression-free survival (PFS), and patients with VEGF levels less than the median had longer PFS and OS when treated with bevacizumab, suggesting that antiangiogenic therapy could benefit subgroups of MPM patients (Karrison *et al*, 2007). Several studies of bevacizumab as well as other new biological agents in combination with pemetrexed and platinum are ongoing (http://www.clinicaltrials.gov). However, the following points should be considered: (1) targeted therapy in combination with cytoreductive chemotherapy should not be given to all patients irrespective of their characteristics, but only to individuals presenting the molecular target of the therapy and in whom these targets are crucial for cancer cell survival (Gutierrez *et al*, 2009) and (2) the prospects of integrating targeted agents with cytotoxic drugs should include the search of optimal cytoreduction based on molecular mechanisms underlying synergistic drug interaction (Giovannetti *et al*, 2008). Therefore, preclinical studies investigating new combinations of targeted agents with cytotoxic chemotherapy and deciphering the molecular mechanisms of drug interaction as well as potential markers of drug activity are urgently needed.

The aim of the current study was to evaluate the molecular and cellular characteristics underlying the synergistic cytotoxicity observed between new targeted agents and the carboplatin–pemetrexed combination through an *in vitro* assessment of their pharmacologic interaction. We found a potent synergistic interaction between carboplatin–pemetrexed and the EGFR/VEGFR-2/RET inhibitor vandetanib against four human MPM cell lines. Several factors including apoptosis induction, modulation of phosphorylation and expression of critical gene products involved in drug activity contributed to this synergistic interaction. Their expression was also validated in a panel of MPM biopsies and might therefore lead to the selection of individual patients for personalised therapies.

## Materials and methods

### Cell lines and drugs

Four human MPM cell lines (Table 1), NCI-H28 and NCI-H2052 (sarcomatoid), NCI-H2452 (epithelioid) and MSTO-211H (biphasic), were obtained from ATCC (Manassas, VA, USA) and tested within the last 6 months by morphology check and growth curve analysis according to the Cell Line Verification Recommendations (ATCC-Technical-Bulletin#8, 2008).

Cells were cultured in DMEM, containing 2 mM L-glutamine, supplemented with 10% heat-inactivated fetal bovine serum, and 1% penicillin–streptomycin (10 000 U ml^–1^), at 37 °C under an atmosphere of 5% CO_2_. The cells were maintained in 75 cm^2^ culture flasks (Greiner-Bio-One, Frickenhausen, Germany) and harvested with trypsin-EDTA (Invitrogen, Paisley, UK) at their exponential phase of growth phase.

*In vitro* studies were carried out using the drugs detailed in Table 2.

### Analysis of mutations and polymorphisms in determinants of drug activity

The MPM cells were characterised for *EGFR* and *k-Ras* mutations. Furthermore, we evaluated the *AKT1-SNP4-G/A* (rs#1130233) polymorphism, related to gefitinib resistance (Giovannetti *et al*, 2010), as well as polymorphisms that may influence pemetrexed sensitivity, such as the *methylenetetrahydrofolate reductase* (*MTHFR*) *MTHFR-C677T* (rs#1801133) and the *reduced folate carrier* (*RFC*) *RFC-G80A* (rs#1051266), and carboplatin activity, such as the DNA repair systems *excision-repair cross-complementing-group-1* (*ERCC1*), and *xeroderma-pigmentosum group-D* (*XPD*) polymorphisms *ERCC1-C118T* (rs#11615), *XPDAsp312Asn* (rs#1799793) and *XPDLys751Gln* (rs#13181). DNA was isolated using miniDNA-kit (Qiagen, Hilden, Germany). DNA yields and integrity were checked at 260–280 nm with NanoDrop-1000-Detector (NanoDrop-Technologies, Wilmington, DE, USA). Nested PCR to amplify *EGFR* (exons 18–21) and *K-Ras* (exons 1–2) and sequencing of PCR products on ABI-3100 genetic analyser (Applied Biosystems, Foster City, CA, USA) was performed as described (Giovannetti *et al*, 2010). All polymorphisms were evaluated with Taqman-probes-based assays using the ABIPRISM-7500HT instrument (Applied Biosystems). These PCR reactions were performed using 20 ng of DNA (Giovannetti *et al*, 2010).

### Cytotoxicity assays

Cell growth inhibitory effects of the drugs (0.001–50 *μ*M, 72-h exposure) were studied using MTT and SRB assays. For this purpose, cells were plated at 5 × 10^4^ cells per well and growth inhibition was expressed as the percentage of control (vehicle-treated cells) absorbance (corrected for absorbance before drug addition). The 50% inhibitory concentration of cell growth (IC_50_) was calculated by non-linear least squares curve fitting (GraphPad PRISM, Intuitive Software for Science, San Diego, CA, USA).

### Drug combination studies

Several studies showed a more efficient interaction when cytotoxic agents are given before or simultaneously with EGFR-TKIs (Morelli *et al*, 2005; Bianco *et al*, 2006; Van Schaeybroeck *et al*, 2006; Giovannetti *et al*, 2008). Therefore, combination studies were focused on simultaneous treatment with the compounds used in the clinical setting (i.e., pemetrexed and carboplatin, alone and in combination) and vandetanib, the most potent targeted compound in the previous cytotoxicity studies. Vandetanib was also chosen for its potential to affect several molecular mechanisms that might sensitise cells to the cytotoxic activity of pemetrexed and carboplatin (e.g., apoptosis induction, and modulation of key drug determinants), as assessed in the experiments detailed below.

Each combination was tested in at least six different concentrations, using a constant ratio calculated with respect to drug IC_50_s. The cytotoxicity of these combinations was compared with the cytotoxicity of each drug alone using the combination index (CI), where CI<0.9, CI=0.9–1.1 and CI>1.1 indicated synergistic, additive and antagonistic effects, respectively (Bianco *et al*, 2006). Data analysis was carried out using CalcuSyn software (Biosoft, Oxford, UK).

### Cell-cycle analysis

Cell-cycle modulation induced by treatments at IC_50_s for 72 h was studied by propidium iodide (PI) staining using a FACScan (Becton Dickinson, San José, CA, USA). Data analysis was carried out with CELLQuest (Becton Dickinson), while cell-cycle distribution was determined using Modfit software (Verity-Software, Topsham, ME, USA).

### Evaluation of apoptosis

Single drugs and their combinations were also characterised for their ability to induce apoptosis, as detected after 72-h drug exposure at IC_50_s. Apoptosis was determined with the FITC-AnnexinV-Apoptosis kit (Becton Dickinson) according to the manufacturer's protocol. Cells were stained by the addition of both 5 *μ*l AnnexinV-FITC and 10 *μ*l PI solution. The samples were analysed with a FACScan and data analysis was carried out with FACSdiva software (Becton Dickinson).

Further studies were performed with bisbenzimide-HCl staining, as described (Bianco *et al*, 2006).

### RT–PCR

Gene expression of the following key determinants of drug sensitivity was assessed by RT–PCR: *EGFR* (NM_005228.3), *VEGFR-2* (NM_002253.2), *RET* (NM_020363.4) *ERCC1* (NM_001983), *XPD* (NM_000400), *thymidylate synthase* (*TS*; NM_0010711), *dihydrofolate reductase* (*DHFR*; NM_000791), *glycinamide ribonucleotide formyltransferase* (*GARFT*; NM_000819), *RFC* (NM_194255.1) and *folyl-polyglutamate synthetase* (*FPGS*; NM_004957). Since previous studies reported a strong correlation between expression levels of TS and its upstream transcriptional regulator E2F-1 (Huang *et al*, 2007), and other studies showed that EGFR-TKIs affected E2F-1 (Kobayashi *et al*, 2006; Suenaga *et al*, 2006), we also evaluated the expression of *E2F-1* (NM_005225.2).

Conversely, we did not evaluate the expression of the folate receptor *α* (FR*α*), which was not correlated with the sensitivity to pemetrexed in MPM cells and samples (Nutt *et al*, 2010).

However, we studied whether single drugs and their combinations, at IC_50_s, modulated expression of *ERCC1*, *XPD*, *TS* and *E2F-1*. RNA was extracted by the QiaAmp RNA mini-Kit (Qiagen), and reverse transcribed. Primers and probes were obtained from Applied Biosystems (Giovannetti *et al*, 2008; Nannizzi *et al*, 2010).

Amplification data were normalised to *β*-actin, and quantification of gene expression was performed using standard curves obtained with dilutions of cDNA from Quantitative-PCR Human-Reference Total-RNA (Stratagene, La Jolla, CA, USA). Results of basal expression analysis of single or combined genes (e.g., *FPGS* × *RFC*/(*TS* × *DHFR* × *GARFT*)) were related to chemosensitivity, while quantification of gene expression in treated cells was performed using the ΔΔ*C*_T_ calculation, where *C*_T_ is the threshold cycle. The amount of target gene, normalised to *β*-actin, and relative to the calibrator (untreated cells), was expressed as 2^−ΔΔ*C*_T_^ and reported as percent variation.

### Western blot analysis

Single drugs and their combinations, at IC_50_s, were also studied for their ability to modulate protein expression of TS. After 72-h exposure, cell pellets were collected and lysed, and 20 *μ*g of proteins were separated on SDS–PAGE gel, followed by blotting onto a nitrocellulose membrane. The membrane was pre-incubated in blocking buffer (0.5% milk powder, 0.5% BSA in TBS-T) for 1 h, while the primary polyclonal TS antibody (1 : 1000, kindly provided by Dr GW Aheme) was added overnight, at 4 °C. After washing in TBS-T, the blots were incubated for 1 h with anti-rabbit horseradish peroxidase-labelled secondary antibodies (1 : 2000, DakoCytomation, Glostrup, Denmark). Antibody binding was detected using enhanced chemoluminescence. Densitometric analysis of the images captured on the VersaDoc3000 instrument (Bio-Rad, Philadelphia, PA, USA) was performed with the Kontron-Analysis-Image software (Kontron-Electronik, Munich, Germany).

### Evaluation of TS *in situ* activity

To evaluate the possible modulation of TS activity, we determined its potential inhibition in intact cells, after 24-h drug exposure at IC_50_s. For this purpose, cells were plated at 0.25 × 10^6^ cells in six-well plates. After 22 h of drug treatment, (5-3H)-deoxycytidine (0.3 *μ*M, final specific activity 1.6 Ci mmol^–1^) was added. After uptake, (5-3H)-deoxycytidine was phosphorylated to (5-3H)-dCMP, which was deaminated to (5-3H)-dUMP, which was in turn methylated to dTMP releasing 3H_2_O. Production of 3H_2_O was measured by collecting medium samples after 2 h and counting of the radioactivity as described (Giovannetti *et al*, 2008).

### EGFR, ERK1/2 and Akt ELISA phosphorylation assays

To study the effect of drug treatments on the activation of EGFR, as well as of ERK1/2 and Akt, MPM cells were exposed for 2 h to IC_50_s of single drugs and their combinations after pulse stimulation with EGF (10 ng ml^–1^), as described (Janmaat *et al*, 2006). After protein extraction from cell pellets, EGFR phosphorylation at the tyrosine residue at position 1173 (EGFR [pY1173]), dual phosphorylation of ERK2 at threonine-185 and tyrosine-187 (ERK2 [pTpY185/187]) and ERK1 at threonine-202 and tyrosine-204 (ERK1 [pTpY202/204]) and Akt phosphorylation at serine-473 (Akt [pS473]) were evaluated with specific ELISA assays (Invitrogen), and normalised, respectively, to the total EGFR, ERK1/2 and Akt, as well as to protein content (Bianco *et al*, 2006).

### ELISA measurement of VEGF levels

Measurement of VEGF levels in medium was performed after exposing 1 × 10^5^ cells to IC_50_ concentrations of the drugs, alone or in combination. Samples of the medium (200 *μ*l) were taken after 24 h, centrifuged for 20 min at 1000 **g** and VEGF levels were measured using a specific kit (R&D Diagnostics, Minneapolis, MN, USA). A calibration line was included in each plate.

### PCR and immunohistochemistry in MPM tissues

RNA was isolated from 44 MPM paraffin-embedded sections using the RecoverAll-Total-Nucleic-Acid Isolation kit (Ambion, Applied Biosystems) from chemonaive patients. RNA quality was checked by 260–280 nm measurements and PCR analysis of *TS* and *EGFR* was performed as described above. Immunohistochemical studies were carried out using specific monoclonal antibodies for EGFR (Neomarkers, Union City, CA, USA; diluted to a final concentration of 4 *μ*g ml^–1^), and TS (clone 106, dilution 1 : 100; Dako). To enhance the immunoreactivity, standard 2 *μ*m thick sections were submitted to antigen retrieval after deparaffination and rehydration. The endogenous peroxidase activity was blocked with 3% hydrogen peroxidase for 30 min, and the primary antibody incubation was carried out for 1 h at room temperature. Negative controls were obtained replacing the primary antibodies with buffer. The immune reaction was revealed with a biotin-free detection system based on a dextran chain linked to the secondary antibody and peroxidase (EnVision, DakoCytomation). Staining was performed with 3,3′-diaminobenzidine (DAB; DakoCytomation) as a chromogen and sections were counterstained with haematoxylin. Similar to previous studies (Destro *et al*, 2006; Zucali *et al*, 2011a, 2011b), the results were interpreted using a system based on staining intensity and on the number of stained cells. Endothelial cells from tonsils served as external positive controls, whereas lymphocytes were used as intra-tumoural positive control.

### Statistical analysis

All experiments were performed in triplicate and repeated at least twice. Data were expressed as mean values±s.e. and analysed by Student's *t*-test or ANOVA followed by the Tukey's multiple comparison test. The Pearson/Spearman correlation and regression analysis were used to demonstrate the relationship between gene expression profile and chemosensitivity, as well as between erlotinib and gefitinib cytotoxic activity, TS mRNA and protein expression and modulation of *E2F-1* and *TS* mRNA expression. The level of significance was *P*<0.05.

## Results

### Genetic background of the human MPM cell lines

Genomic DNA and RNA extracted from the various MPM cells were used to detect mutations, polymorphisms and mRNA expression levels of genes potentially affecting drug activity, as depicted in Table 1. No mutations were detected in *EGFR* and *Ras*, as previously reported in the Cosmic data bank for cells and mesothelioma patients (http://www.sanger.ac.uk/genetics/CGP/cosmic/).

### Growth inhibition studies and correlation with genetic background

A dose-dependent inhibition of tumour cell growth was observed with all drugs studied using four established MPM cell lines (Table 2). The IC_50_ values ranged from 0.021±0.003 (pemetrexed in MSTO-211H cells) to 14.59±4.58 *μ*M (sorafenib in H2452 cells), and most compounds (gefitinib, erlotinib, enzastaurin and ZM447439) displayed similar growth inhibitory activities in these tumour cell lines. A strong correlation was found between cellular sensitivity to erlotinib and gefitinib (*R*^2^=0.981, *P*=0.018).

MSTO-211H cells harbouring the *XPD-Asp312Asn/Lys751Gln* genotypes were the most drug-sensitive cells, whereas H28 cells bearing the *XPD-Asp312Asp/Lys751Lys* genotypes were the most inherently drug resistant to both carboplatin and pemetrexed. However, no clear relationship could be established between polymorphisms and chemosensitivity.

Basal mRNA levels of *ERCC1* and *XPD*, as well as *TS*, *DHFR*, *GARFT*, *FPGS* and *RFC* were also evaluated for their possible correlation with carboplatin and pemetrexed sensitivities, respectively.

The lower chemosensitivity of the H28 cells to pemetrexed is likely related to the higher expression levels of genes encoding *TS* and *GARFT*, relative to other tumour cell lines. In addition, this cell line displayed a low level of RFC expression, which may be associated with a diminished uptake of pemetrexed, despite an intermediate expression of *FPGS*. In contrast, drug-sensitive NCI-H2052 cells exhibited high *RFC* and *FPGS* expression but low *TS* levels. However, when statistically examined individually, none of the target genes was found to be related to cell sensitivity, whereas a good correlation was observed between the ratio of *FPGS* × *RFC*/(*TS* × *DHFR* × *GARFT*) and pemetrexed IC_50_ values (*R*^2^=0.928; *P*=0.020). The targeted cytotoxic compound displaying the most potent growth inhibitory activity among the four cell lines was vandetanib (IC_50_ range, 0.32±0.07 (H28) to 3.52±1.13 *μ*M (H2452)), which was therefore further evaluated in combination with carboplatin and pemetrexed.

### Pharmacological interaction

Since the CI method recommends a ratio of concentrations at which drugs are equipotent, combination studies were performed using fixed ratios with IC_50_ values for carboplatin and pemetrexed. Experiments were performed in H28 and H2452 cells, which were relatively resistant to both the antiproliferative effects of carboplatin and pemetrexed, but characterised by the lowest and the highest IC_50_ value for vandetanib, respectively. Furthermore, although two cell lines cannot represent the heterogeneity and complexity seen in human MPM, the two cell lines included in these studies were representative of the most common hystotypes (epithelioid and sarcomatoid), comprising >85% of all MPM cases, as well as of the prognostic extremes.

**Figure 1:**
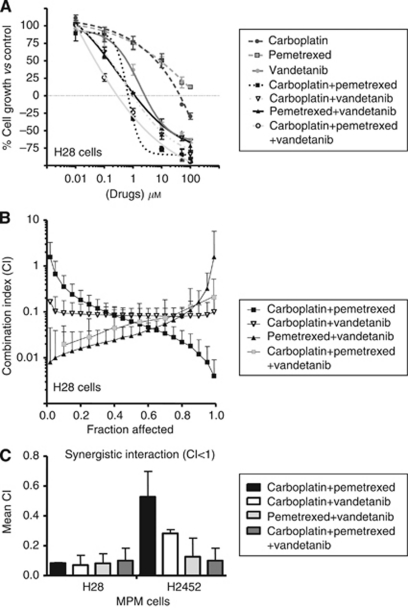
Cytotoxicity and pharmacological interaction of carboplatin, pemetrexed and vandetanib. (**A**) Representative curves of growth inhibitory effects of carboplatin, pemetrexed, vandetanib and their combinations in H28 cells (for the combinations drug concentrations on the *X*-axis are referred to pemetrexed). (**B**) CI fraction affected (FA) plots of the carboplatin–pemetrexed, carboplatin–vandetanib, pemetrexed–vandetanib and carboplatin–pemetrexed–vandetanib combinations in H28 cells. (**C**) Mean CI values of all the combinations in H28 and H2452 cells. CI values at FA of 0.25, 0.5, 0.75 and 0.9 were averaged for each experiment, and this value was used to calculate the mean between experiments, as described in the Materials and Methods section. Points and columns, mean values obtained from three independent experiments; bars, s.e.m.

For the triple simultaneous combination, we used vandetanib at a fixed IC_25_ concentration. Both the carboplatin and vandetanib combinations reduced the IC_50_ values of pemetrexed in the studied cell lines. Similarly, vandetanib significantly reduced the IC_50_ values of carboplatin, and the triple combination of carboplatin, pemetrexed and vandetanib displayed the most potent cytotoxic effect. Representative growth inhibition curves for H28 cells are shown in Figure 1A. Multiple drug-effect analysis revealed strong synergistic effects in all the treatments. For example, the CI plots (Figure 1B) in H28 cells showed a clear synergistic interaction at the more relevant FA values in all the three doublet combinations (⩾25%). The average CI values for all the combinations in the two MPM cell lines are summarised in Figure 1C. To explore the mechanisms underlying these synergistic drug interactions, we performed several biochemical analyses (cell-cycle analysis, apoptosis detection, western blot analysis), as detailed below. Most of these analyses were performed after 72-h drug exposure, while the phosphorylation of EGFR, ERK1/2 and Akt was evaluated after 2 h, in agreement with previous studies showing that the dynamics of protein phosphorylation was optimally detected at early time points (Janmaat *et al*, 2006). Similarly, the measurement of VEGF and TS levels was performed after 24-h exposure, which represents the optimal exposure time for these assays (Tekle *et al*, 2008).

### Cell-cycle alterations and induction of apoptosis

Flow cytometric DNA analysis was performed to evaluate the effect of carboplatin, pemetrexed, vandetanib and their combinations on cell-cycle distribution and to determine whether or not their cell-cycle alterations might provide clues to optimise drug scheduling. All these agents were able to affect the cell-cycle parameters of all four MPM cell lines studied (Table 3).

Carboplatin induced a slight cell-cycle arrest in the G_2_-phase in all cell lines, ranging between +4.7% and +7.9% in MSTO-211H and H28 cells, respectively. In contrast, pemetrexed increased the fraction of cells in the S-phase; this increment was most pronounced in MSTO-211H and H2052 (1.6- and 1.9-fold, respectively). Vandetanib increased the fraction of cells at the G_1_-phase, ranging from +4.3% to 13.5% in H28 and MSTO-211H cells, respectively. Remarkably, all combinations markedly increased the S- and G_2_-phase population compared with controls. In particular, carboplatin+vandetanib combination resulted in a lower increase in S-phase population, since more cells accumulated in G_2_-/M-phase. In contrast, all the combinations including pemetrexed mostly increased the percentage of cells in S-phase.

All treatments induced cell death, as shown by the presence of a cell population with sub-G_1_ DNA content in the FACS analysis, which was confirmed by AnnexinV assay and fluorescence microscopy analysis of typical apoptotic morphology after staining with the intercalating DNA dye bisbenzimide. The combinations of two drugs significantly increased the apoptotic index with respect to controls, up to 17.3% in MSTO-211H cells after pemetrexed+vandetanib exposure, and the triple combination was more active and caused a significant induction of apoptosis when compared with both controls and pemetrexed-treated or carboplatin-treated cells in all cell lines.

### EGFR, ERK1/2 and Akt phosphorylation assays

**Figure 2:**
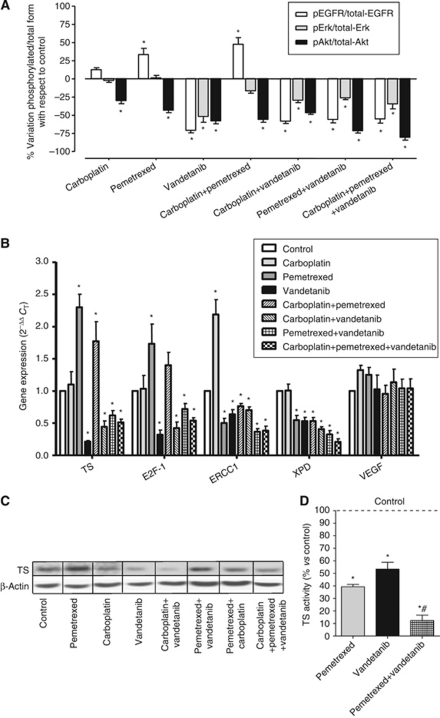
Effects of carboplatin, pemetrexed, vandetanib and their combinations on critical determinants of drug activity in H28 cells. (**A**) Modulation of phosphorylation of EGFR and downstream molecules ERK1/2 and Akt. (**B**) Modulation of *TS*, *E2F-1*, *ERCC1*, *XPD* and *VEGF* mRNA as determined by real-time RT–PCR. (**C**) Representative blots illustrating the modulation of TS protein expression. (**D**) Modulation of TS activity. Columns, mean values obtained from three independent experiments; bars, s.e.m. ^*^Significantly different from controls (*P*<0.05). ^#^Significantly different from pemetrexed-treated cells (*P*<0.05).

The analysis of the EGFR pathway was performed in H28 and H2452 cells. Expectedly, vandetanib significantly suppressed EGFR phosphorylation at the tyrosine residue pY1173, with percentages of reduction of phospho-EGFR ranging up to −74.3% in H28 cells. Conversely, pemetrexed enhanced pY1173-EGFR levels. The pemetrexed+carboplatin combination also significantly increased pY1173-EGFR levels, whereas all drug combinations including vandetanib reduced the phosphorylation status of EGFR, albeit to a lower extent than vandetanib alone (Figure 2A).

Since EGFR signalling is transduced mainly through the Akt and ERK1/2 kinase pathways, we also investigated the phosphorylation status of Akt and ERK1/2. Phospho-ERK1/2 levels were markedly reduced (>50%) by vandetanib in H28 cells, while a lower degree of inhibition (∼20%) was detected in H2452 cells. In contrast, pemetrexed and carboplatin exposure had a negligible effect on ERK1/2 phosphorylation, while a slight reduction of phospho-ERK1/2 was detected with all drug combinations. Akt phosphorylation was significantly decreased (>50%) by vandetanib in H28 cells, whereas the inhibition was of about 35% in H2452 cells. Similar results were obtained after exposure to both pemetrexed and carboplatin. Akt phosphorylation was additionally reduced by the simultaneous combination, with a degree of inhibition up to −80.4% and −65.7% after the triple combination of carboplatin+pemetrexed+vandetanib in H28 and H2452 cells, respectively.

### Modulation of TS and E2F-1 mRNA and TS protein expression

To gain further insight into the molecular mechanisms involved in regulating drug interactions, we examined alterations in the expression of TS, the primary target of pemetrexed. H2452 and H28 cells treated with vandetanib had a 2.3- and 4.8-fold decrease in *TS* mRNA expression (Figure 2B). In contrast, pemetrexed significantly increased *TS* mRNA expression in both cell lines. However, all drug combinations including vandetanib, not only prevented the pemetrexed-induced increase, but rather decreased TS expression. Similarly, both vandetanib and its combinations significantly reduced *E2F-1* mRNA levels in MPM cells, and the modifications in *TS* and *E2F-1* expression levels after vandetanib and vandetanib combinations were correlated (*R*^2^=0.87).

Thymidylate synthase expression was also studied at the protein level using western blot analysis in H2452 and H28 cells (Figure 2C). This analysis revealed a marked induction in pemetrexed-treated H2452 cells, while vandetanib repressed TS expression, with barely detectable levels observed in protein extracts isolated from H28 cells. A reduction in TS expression was also detected in cells treated with cytotoxic drug combinations containing vandetanib.

### Modulation of TS activity

Since protein expression of TS is not necessarily predictive of the cellular catalytic activity of TS, we hence evaluated TS activity by the TS *in situ* assay, in which intact H28 (Figure 2D) and H2452 cells were used. This assay showed a similar inhibition of TS by pemetrexed and vandetanib in both cell lines. Most interestingly, the combination of pemetrexed and vandetanib almost completely blocked TS activity, and statistical analysis revealed a significant reduction with respect to that observed after pemetrexed exposure (*P*<0.037 and *P*<0.027 in H28 and H2452 cells, respectively).

### Alterations of ERCC1 and XPD mRNA expression

Drug-treatment-dependent alterations in gene expression levels of *ERCC1* and *XPD* in H28 cells are shown in Figure 2B. Carboplatin had a negligible effect on *XPD* expression, but caused a significant increase in *ERCC1* levels, up to 2.2- and 2.0-fold in H28 and H2452 cells, respectively. In contrast, pemetrexed significantly reduced *ERCC1* in both cell lines. Pemetrexed also decreased *XPD* expression, up to −62.6% in H2452 cells. Similarly, vandetanib significantly decreased both *ERCC1* and *XPD* expression. Finally, *ERCC1* and *XPD* levels were reduced by all drug combinations in both cell lines.

### Modulation of VEGF expression

Since vandetanib has been reported to have an impact on plasma VEGF levels, we evaluated the expression of VEGF levels in both cells and culture medium. Vandetanib neither affected VEGF mRNA levels nor VEGF secretion into the growth medium. On the other hand, pemetrexed and carboplatin induced a slight increase in VEGF mRNA expression (Figure 2B) and VEGF protein in the cell culture medium (data not shown). However, all combinations had a negligible effect on VEGF expression in both cell lines (data not shown).

### Expression of EGFR and TS in specimens from MPM patients

**Figure 3:**
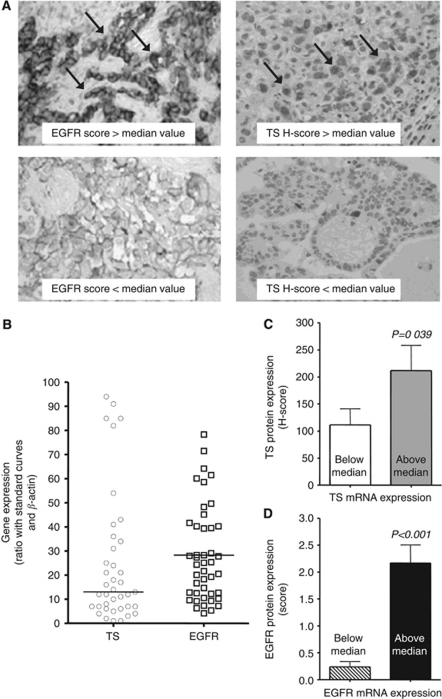
Immunohistochemistry and PCR of MPM samples. (**A**) Representative examples (original magnification, × 40) from staining of paraffin-embedded MPM samples (36 epithelioid, 7 biphasic and 1 sarcomatoid) for TS (right panels) and EGFR (left panels). TS showed positive cytoplasmic and nuclear staining in most tissue sections (arrows), with intense staining in 8 out of 44 samples, whereas EGFR membrane immunoreactivity (arrows) was detected in 23 out of 44 cases (52.3%): 15.9% specimens showed high expression levels. (**B**) mRNA expression values of TS and EGFR observed across the cohort of MPM patients, and correlation between TS (**C**) and EGFR (**D**) mRNA and protein expression. Values of mRNA and protein expression were calculated as described in the Materials and Methods section. Data were analysed by both Student's *t* and *χ*^2^-test. Columns, mean values; bars, s.d.

Epidermal growth factor receptor and TS mRNA and protein expression was detectable in most tumour specimens. Representative results from staining of paraffin-embedded MPM tissue samples for EGFR, and TS are shown in Figure 3A; the expression values observed across the cohort of patients subjected to transcript level analysis (Figure 3B), were similar to the expression levels observed in the MPM cell lines. A significant correlation was found between mRNA and protein levels (Figure 3C), whereas no statistical associations were found between EGFR and TS, as well as between EGFR and TS levels and tumour histology/stage/grade.

## Discussion

The present study demonstrates that vandetanib is the targeted antitumour agent displaying the most potent growth inhibitory effects in a panel of human MPM cell lines characterised by distinct molecular properties. Limited published preclinical research focusing on this issue reported similar cytotoxic activity in H28, MSTO-211H and H226 MPM cell lines (Nutt *et al*, 2009), as well as in EHMES-10 MPM cells harbouring a RET/PTC3 rearrangement (Ogino *et al*, 2008). Sensitivity to vandetanib in the MPM cell lines also fell within the range of IC_50_ values previously reported for other carcinoma cell lines, including pancreas, breast, colon, gastric and ovarian cancer cells with functional EGFR but lacking VEGFR-2 (Ciardiello *et al*, 2003; Bianco *et al*, 2006). Furthermore, vandetanib interacted synergistically with carboplatin, pemetrexed and their combination, and increased the apoptotic fraction induced by the carboplatin–pemetrexed combined treatment. These results are in agreement with previous studies, showing enhanced apoptotic cell death after combined treatment with vandetanib and paclitaxel, docetaxel, gemcitabine or oxaliplatin in several cancer cell lines (Morelli *et al*, 2005; Yokoi *et al*, 2005; Bianco *et al*, 2006).

**Figure 4:**
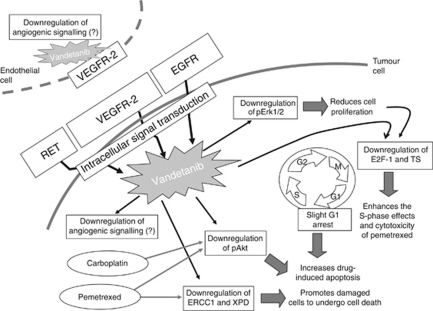
Molecular signalling pathways and key determinants involved in the synergistic interaction of vandetanib with pemetrexed and carboplatin. Vandetanib enhanced the growth inhibitory effects of cytotoxic drugs through its pronounced antisignalling effects downstream of EGFR. Furthermore, the modulation of TS and DNA repair genes promotes pemetrexed and carboplatin activity.

A phase II trial showed that non-small cell lung cancer (NSCLC) patients receiving the triple combination of vandetanib, carboplatin and paclitaxel had longer PFS compared with the control arm of paclitaxel+carboplatin (Heymach *et al*, 2008), while, in the phase III ZEAL study, randomising 534 NSCLC patients to pemetrexed+vandetanib or pemetrexed+placebo as second-line treatment, the addition of vandetanib failed to show an improvement in PFS, but significantly improved RR (de Boer *et al*, 2011). To the best of our knowledge, no clinical trials are currently evaluating the addition of vandetanib to the carboplatin–pemetrexed regimen and hence no predictive biomarkers for clinical outcome for this combination have been identified to date in MPM. Therefore, the present study was also aimed at investigating the molecular mechanisms underlying the synergistic interaction with pemetrexed and carboplatin and exploring possible determinants/biomarkers of drug activity (Figure 4).

By targeting the EGFR-dependent cancer cell proliferation and the VEGFR-2-dependent tumour angiogenesis pathways, vandetanib offers the potential advantages of blocking two key pathways in different tumour types, namely cell proliferation and angiogenesis. Several studies demonstrated that MPM is characterised by dysregulation of molecular mechanisms involved in cell proliferation and angiogenesis (Zucali *et al*, 2011a, 2011b). Malignant pleural mesothelioma has one of the highest VEGF levels among solid tumours (Linder *et al*, 1998), VEGFR-2 expression is detected in most MPM samples (Nutt *et al*, 2009), and an increased vascularisation is a poor prognostic factor (Edwards *et al*, 2003). Therefore, small molecule inhibitors that target VEGFR-2 in both vessels and tumour cells, such as vandetanib, may be useful for MPM treatment. However, in agreement with previous findings (Nutt *et al*, 2009), VEGFR-2 was not detectable in H28 cells, which were the most sensitive to vandetanib, suggesting that other pathways have a crucial role for vandetanib cytotoxicity. Moreover, vandetanib neither affected *VEGF* mRNA expression nor VEGF secretion into the medium in our MPM cells, reflecting the transient, inconsistent and relatively modest changes in plasma VEGF after vandetanib treatment in patients (Hanrahan *et al*, 2010). Similarly, RET expression was not detectable in H28 and MSTO-211H cells, suggesting that the modulation of this kinase is not involved in drug activity in MPM cells lacking RET oncogenic rearrangement, as described previously (Ogino *et al*, 2008). Therefore, the activity of vandetanib seems limited to the inhibition of EGFR and associated pathways. These findings are in agreement with previous preclinical studies, showing a prominent activity of EGFR inhibitors in mesothelioma cells (Nutt *et al*, 2009; Stoppoloni *et al*, 2010; Barbieri *et al*, 2011). In particular, in a study by Nutt *et al* (2009), which explored the effects of five TKIs, vandetanib displayed the lowest IC_50_ values across all the three MPM cell lines tested, including one cell line that was positive for VEGFR-2 expression.

Epidermal growth factor receptor is a key driver of cell proliferation, which was detected in all the cell lines and in the majority of mesothelioma specimens in the present study, as well as in previous studies (Destro *et al*, 2006; Gaafar *et al*, 2010). Vandetanib significantly reduced EGFR phosphorylation, while pemetrexed exposure resulted in a significant increase in phospho-EGFR levels. These results are in agreement with previous data, showing a schedule-dependent synergism of pemetrexed and the EGFR-TKI erlotinib associated with pemetrexed-induced EGFR phosphorylation (Li *et al*, 2007; Giovannetti *et al*, 2008). Similar effects were observed for other cytotoxic drugs in NSCLC cells displaying a synergistic interaction with gefitinib, as well as with the TS-inhibitor 5-fluorouracil (5-FU) in colorectal cancer cells (Van Schaeybroeck *et al*, 2005, 2006). In addition to the impact on EGFR phosphorylation, downstream mediators of the EGFR signalling pathway were affected not only by vandetanib but also by pemetrexed and carboplatin, which reduced Akt phosphorylation. The Akt signalling pathway is frequently activated in MPM and its inhibition increases drug sensitivity (Altomare *et al*, 2005). Previous studies showed conflicting results regarding the modulation of phospho-Akt levels by pemetrexed (Li *et al*, 2007; Giovannetti *et al*, 2008), which may be related to the discrepancy between drug exposure conditions and different sensitivities of experimental methods. Our findings are in agreement with studies, demonstrating reduced Akt phosphorylation after exposure to the antimetabolite gemcitabine (Bianco *et al*, 2006; Feng *et al*, 2007). In particular, Feng *et al* (2007) proposed a model of gemcitabine-induced apoptosis via EGFR degradation, hence providing a plausible mechanism by which a cytotoxic compound may affect EGFR levels and Akt signalling pathways. Drug treatment through c-Src activation leads to EGFR phosphorylation, which promotes receptor ubiquitination. Epidermal growth factor receptor is then targeted to lysosomes for degradation, thereby resulting in downregulation of phospho-Akt. Since phospho-Akt regulates antiapoptotic mechanisms and previous *in vitro* studies showed that its downregulation by the pemetrexed–erlotinib combination correlated with the enhancement of apoptosis and antitumour activity in lung cancer cells (Giovannetti *et al*, 2008), the reduction of phospho-Akt may explain the increased apoptosis after pemetrexed–vandetanib and carboplatin–vandetanib combinations in MPM cells.

The increased induction in apoptosis after the carboplatin–vandetanib combination may also be related to DNA damage, which was reported to be important for the efficacy of the combination of EGFR-TKIs with different cytotoxic compounds, including platinum derivatives (Morelli *et al*, 2005). Damage induced by chemotherapy can convert EGFR ligands from growth factors into survival factors for cancer cells that express functional EGFR. In this context, the simultaneous blockade of EGFR signalling after exposure to a cytotoxic drug such as carboplatin could cause non-repairable damage thereby leading to apoptosis.

In addition to the effects on signalling pathways, the present study shows that vandetanib also interfered with the cytotoxic activity of pemetrexed and carboplatin. Indeed, vandetanib significantly inhibited TS, the expression of which has been recently correlated with outcome after pemetrexed-based regimens in MPM (Righi *et al*, 2010; Zucali *et al*, 2011a, 2011b). These data are in accord with the previous observations that antitumour agents other than antifolates and 5-FU also modulate TS levels.

As an RNA-binding protein, TS protein regulates its own synthesis by blocking the translation of its own mRNA, and its binding to a specific inhibitor leads to upregulation of TS protein (Chu *et al*, 1991). In agreement with this hypothesis, as well as with the observed increase in *TS* mRNA expression, as previously detected after treatment with pemetrexed and 5-FU (Peters *et al*, 2002; Giovannetti *et al*, 2008), TS protein expression in cell extracts was enhanced after pemetrexed exposure. However, other studies reported that vinorelbine and irinotecan suppressed TS expression, hence favouring the activity of 5-FU (Guichard *et al*, 1998; Matsumoto *et al*, 2004). Thymidylate synthase expression and activity was also decreased by EGFR-TKIs, thereby displaying synergistic interaction with 5′-deoxy-5-fluorouridine (Magné *et al*, 2003; Budman *et al*, 2006). In the latter study, it was postulated that downregulation of TS was related to decrease in S-phase and increase in G_1_-phase. However, the marginal increase in G_1_-phase observed in the present study does not fit with this hypothesis, and the effects on mRNA and protein expression suggest that TS alterations are mediated by mechanisms involving transcriptional regulation. In this regard, we detected a reduction of *E2F-1* mRNA expression after vandetanib exposure. These results may be related to the nuclear effects of EGFR, which affect the activity of several cell-cycle proteins and transcription factors, including E2F-1 (Lo and Hung, 2006). High levels of free E2F-1 upregulate the transcription of several proliferation-associated genes including *TS* (Huang *et al*, 2007). Epidermal growth factor receptor-TKIs may affect E2F-1 directly or via downregulation of cyclin D1 (Kobayashi *et al*, 2006; Suenaga *et al*, 2006). In this regard, transfection of gastric cells with a cyclin D1 antisense oligodeoxynucleotide reduced *TS* mRNA and significantly increased 5-FU and methotrexate sensitivity (Shuai *et al*, 2006).

However, the synergistic interaction with carboplatin is likely to be mediated by the downregulation of *ERCC1* and *XPD* expression, which was found after exposure to both vandetanib and pemetrexed. Due to the accelerated rate of DNA replication in neoplastic cells, disruption of folate metabolism and consequent depletion of cellular nucleotide pools causes impaired DNA synthesis and repair. Furthermore, folate deficiency has been shown to act synergistically with alkylating agents to increase DNA strand breaks and mutations as a result of impaired DNA excision repair (Novakovic *et al*, 2006). This effect may depend on the inhibition of gene expression of key enzymes in DNA repair. In particular, our results demonstrated a substantial reduction in the transcript levels of *ERCC1* and *XPD* in all MPM cell lines, as previously detected in a cisplatin-resistant carcinoma cell line treated with 5-FU, as well as in colorectal cancer cell lines treated with pemetrexed (Fujishima *et al*, 1997; Nannizzi *et al*, 2010). Similarly, stimulation of oxaliplatin-DNA adduct formation has previously been correlated with the potentiation of oxaliplatin cytotoxicity by the anti-EGFR cetuximab in HCT-8 colorectal cancer cells. This upregulation was associated with reduced expression of mRNA and protein levels of ERCC1 (Balin-Gauthier *et al*, 2008).

Previous studies showed that cisplatin-induced increases in the expression of ERCC1 following c-*fos*/c-*jun* and iAP-1-binding activity (Rabo *et al*, 1996). In contrast, EGFR inhibition with gefitinib/erlotinib abrogated the *c-fos* mRNA increase after exposure to EGF (Jimeno *et al*, 2006), and EGFR inhibition with gefitinib decreased the upregulation of c-Jun, Fos-B and AP-1 (Dougherty *et al*, 2008). Therefore, one can hypothesise that the inhibition of these transcription factors by vandetanib is involved in the downregulation of *ERCC1*.

In conclusion, the present study demonstrates that vandetanib markedly enhances pemetrexed and carboplatin activity against established MPM cell lines. Furthermore, we have characterised several molecular mechanisms and key determinants associated with these synergistic interactions. Vandetanib significantly reduced EGFR, ERK1/2 and Akt phosporylation, as well as *TS* and *ERCC1*/*XPD* expression, possibly via downregulation of the major transcription factor *E2F-1* and other transcription factors. No pharmacologic activity was found on angiogenic signalling, with VEGFR-2 and RET expression not even detectable in some cell lines, suggesting that similar results might be observed with EGFR inhibitors. However, in agreement with a previous study, vandetanib emerged as the compound with the most potent cytotoxic activity (Nutt *et al*, 2009). Moreover, pemetrexed and carboplatin increased EGFR phosphorylation and reduced Akt phosphorylation, which was further reduced by drug combination, and induced a marked apoptosis. The positive modulation of these key determinants of antitumour drug activity, as well as the detection of their expression in MPM samples, whose variability should be evaluated for possible patients’ stratification in future trials, warrant further preclinical and clinical studies for the rational development of combination chemotherapeutic regimens including vandetanib as an integral component in the treatment of MPM.

## Figures and Tables

**Table 1:** Genetic background of drug activity determinants in MPM cells

	**NCI-H2052**	**NCI-H2452**	**NCI-H28**	**MSTO-211H**
*Polymorphisms*				
*AKT1-SNP4*	*G/G*	*A/A*	*G/G*	*G/G*
*MTHFR-C677T*	*T/T*	*C/T*	*C/C*	*C/T*
*RFC-A80G*	*A/A*	*A/G*	*A/A*	*A/A*
*ERCC1-C118T*	*C/T*	*T/T*	*C/T*	*C/T*
*XPD-Asp312Asn*	*Asp/Asn*	*Asp/Asp*	*Asp/Asp*	*Asp/Asn*
*XPD-Lys751Gln*	*Lys/Gln*	*Lys/Lys*	*Lys/Lys*	*Lys/Gln*
				
*mRNA expression* a				
*EGFR*	30.6±2.6	75.8±6.1	61.9±5.0	45.6±3.3
*VEGFR-2*	0.4±0.1	2.7±1.0	nd	0.9±1.3
*RET*	0.5±0.1	1.1±0.2	nd	nd
*ERCC1*	7.7±0.8	0.3±0.1	0.6±0.1	2.3±0.4
*XPD*	22.4±3.7	11.4±1.9	8.9±1.4	17.5±1.8
*TS*	57.7±5.9	31.3±3.7	106.6±9.4	76.7±6.3
*E2F-1*	18.3±2.1	13.0±0.9	54.3±6.2	25.7±2.8
*DHFR*	48.2±3.9	22.7±1.2	27.5±4.5	21.0±2.0
*GARFT*	1.1±0.1	1.8±0.2	9.8±0.8	3.3±0.5
*RFC*	8.4±2.5	1.3±0.1	1.4±0.3	9.5±0.8
*FPGS*	53.7±5.6	11.1±1.7	23.5±2.3	55.2±1.6

Abbreviations: DHFR=dihydrofolate reductase; EGFR=epidermal growth factor receptor; ERCC1=excision-repair cross-complementing-group-1; FPGS=folyl-polyglutamate synthetase; GARFT=glycinamide ribonucleotide formyltransferase; MPM=malignant pleural mesothelioma; MTHFR=methylene-tetrahydrofolate reductase; nd=not detectable; RFC=reduced folate carrier; TS=thymidylate synthase; Wt=wild-type; XPD=xeroderma-pigmentosum group-D; VEGFR=vascular endothelial growth factor receptor; cDNA=complementary DNA.

a Relative mRNA expression levels were expressed in arbitrary units and normalised to *β*-actin, using a method with standard curves derived from serial dilutions from a reference cDNA obtained from Quantitative-PCR Human-Reference Total-RNA.

**Table 2:** Drugs used and their IC_50_ values (*μ*M) in MPM cells

**Drugs**	**Drug targets**	**H2052**	**H2452**	**H28**	**MSTO-211H**
Carboplatin	DNA	5.30±1.02	2.69±0.71	10.50±2.59	0.51±0.12
Pemetrexed	TS, DHFR, GARFT	0.07±0.01	2.82±0.17	10.96±2.46	0.02±0.01
Vandetanib	EGFR, VEGFR-2/3, RET	1.07±0.04	3.52±1.13	0.32±0.07	1.42±0.03
Sorafenib	Raf, PDGF, VEGFR-2/3	6.09±1.78	14.59±4.58	10.22±1.93	3.18±0.27
Gefitinib	EGFR	5.22±1.53	4.83±1.24	3.99±1.28	4.91±1.04
Erlotinib	EGFR	5.55±1.28	5.26±1.29	3.92±1.06	5.48±1.93
Enzastaurin	PKC*β*, VEGF	11.56±3.82	10.07±2.96	8.11±2.19	10.92±2.59
ZM447439	Aurora kinase B	12.73±3.49	10.40±3.27	11.12±4.02	9.65±3.06

Abbreviations: DHFR=dihydrofolate reductase; DMSO=dimethyl sulfoxide; EGFR=epidermal growth factor receptor; GARFT=glycinamide ribonucleotide formyltransferase; MTT=3-(4,5-dimethylthiazol-2-yl)-2,5-diphenyltetrazolium bromide; PDGF=platelet-derived growth factor; PKC*β*=protein kinase C****β****; RET=rearranged during transfection; SRB=sulforhodamine B; TS=thymidylate synthase; VEGFR=vascular endothelial growth factor receptor; MPM=malignant pleural mesothelioma.

Notes: The drugs were dissolved in DMSO or sterile water and diluted in culture medium before use. IC_50_ concentrations were calculated as mean values±s.e.m. of at least three independent MTT or SRB experiments.

**Table 3:** Cell-cycle modulation and apoptotic index

**Cells**	**Treatment**	**G0/G1 phase (%)**	**S phase (%)**	**G2/M phase (%)**	**Apoptotic index (%)**
NCI-H2052	Control	69.9±4.2	12.8±1.4	17.3±2.0	1.3±0.2
	Carboplatin	64.2±7.1	13.6±0.9	22.2±1.4	5.0±0.4
	Pemetrexed	70.3±6.3	24.5±2.5	5.2±0.2	4.5±0.7
	Vandetanib	78.2±8.5	9.3±0.8	12.5±0.2	6.4±0.2
	Pemetrexed+carboplatin	63.2±6.9	15.8±3.7	21.0±2.5	11.3±1.9^*^
	Pemetrexed+vandetanib	65.7±4.8	17.9±3.2	16.4±1.6	12.6±2.5^*^
	Carboplatin+vandetanib	60.4±5.6	16.8±0.3	22.8±0.9	13.1±3.3^*^
	Pemetrexed+carboplatin+vandetanib	58.8±4.1	19.2±1.1	22.0±3.0	19.4±2.8^**^
NCI-H2452	Control	75.3±7.8	17.3±4.1	7.4±0.5	1.1±0.3
	Carboplatin	72.9±5.3	14.8±1.2	12.3±1.7	4.1±1.2
	Pemetrexed	69.5±6.4	24.0±2.2	6.5±0.4	3.5±0.4
	Vandetanib	80.4±7.2	10.9±1.1	8.7±0.9	3.3±0.5
	Pemetrexed+carboplatin	52.6±4.9	32.8±4.1	14.6±1.1	9.5±2.6^*^
	Pemetrexed+vandetanib	47.0±4.4	37.2±4.0	15.8±1.2	10.7±1.9^*^
	Carboplatin+vandetanib	54.9±5.7	29.6±2.1	15.5±1.0	11.4±2.3^*^
	Pemetrexed+carboplatin+vandetanib	47.2±3.8	37.6±3.1	15.2±0.9	20.5±4.1^**^
NCI-H28	Control	69.9±7.1	12.8±0.3	17.3±1.7	1.3±0.2
	Carboplatin	60.3±5.1	14.5±1.1	25.2±2.2	3.0±1.1
	Pemetrexed	66.2±8.9	16.7±1.1	17.1±1.5	4.5±1.0
	Vandetanib	74.2±4.1	7.5±0.9	18.3±1.6	7.7±1.3
	Pemetrexed+carboplatin	50.0±4.6	28.6±3.1	21.4±2.3	11.2±1.2^*^
	Pemetrexed+vandetanib	42.8±3.5	19.0±2.0	38.2±3.1	13.5±3.3^*^
	Carboplatin+vandetanib	46.1±4.8	14.7±1.3	39.2±2.6	11.1±2.8^*^
	Pemetrexed+carboplatin+vandetanib	51.1±5.7	26.9±3.2	22.0±4.0	18.2±1.8^**^
MSTO-211H	Control	57.5±6.1	19.2±1.1	23.3±3.0	1.5±0.2
	Carboplatin	50.2±5.1	21.8±2.2	28.0±2.9	4.4±0.3
	Pemetrexed	56.1±5.5	31.4±3.0	12.5±1.1	10.1±2.2
	Vandetanib	71.0±6.8	10.7±1.3	18.3±2.1	4.2±0.7
	Pemetrexed+carboplatin	40.3±4.0	34.5±3.1	25.2±2.6	15.1±2.5^*^
	Pemetrexed+vandetanib	28.7±2.6	32.9±4.1	38.4±3.1	17.3±3.1^*^
	Carboplatin+vandetanib	30.3±2.9	28.7±1.3	41.0±0.9	15.9±2.7^*^
	Pemetrexed+carboplatin+vandetanib	27.5±3.1	36.1±4.5	36.4±2.7	26.4±3.3^**^

^*^P<0.05 with respect to control cells, ^**^P<0.05 with respect to pemetrexed+carboplatin-treated cells.

## References

[bib1] Altomare DA, You H, Xiao GH, Ramos-Nino ME, Skele KL, De Rienzo A, Jhanwar SC, Mossman BT, Kane AB, Testa JR (2005) Human and mouse mesotheliomas exhibit elevated AKT/PKB activity, which can be targeted pharmacologically to inhibit tumor cell growth. Oncogene 24: 6080–6089 15897870 10.1038/sj.onc.1208744

[bib2] Balin-Gauthier D, Delord J-P, Pillaire M-J, Rochaix P, Hoffman JS, Bugat R, Cazaux C, Canal P, Allal BC (2008) Cetuximab potentiates oxaliplatin cytotoxic effect through a defect in NER and DNA replication initiation. Br J Cancer 98: 120–128 18182978 10.1038/sj.bjc.6604134 PMC2359709

[bib3] Barbieri F, Würth R, Favoni RE, Pattarozzi A, Gatti M, Ratto A, Ferrari A, Bajetto A, Florio T (2011) Receptor tyrosine kinase inhibitors and cytotoxic drugs affect pleural mesothelioma cell proliferation: insight into EGFR and ERK1/2 as antitumor targets. Biochem Pharmacol; e-pub ahead of print 20 July 2011 10.1016/j.bcp.2011.07.073 21787763

[bib4] Bianco C, Giovannetti E, Ciardiello F, Mey V, Nannizzi S, Tortora G, Troiani T, Pasqualetti F, Eckhardt G, de Liguoro M, Ricciardi S, Del Tacca M, Raben D, Cionini L, Danesi R (2006) Synergistic antitumor activity of ZD6474, an inhibitor of vascular endothelial growth factor receptor and epidermal growth factor receptor signaling, with gemcitabine and ionizing radiation against pancreatic cancer. Clin Cancer Res 12: 7099–7107 17145834 10.1158/1078-0432.CCR-06-0833

[bib5] Budman DR, Soong R, Calabro A, Tai J, Diasio R (2006) Identification of potentially useful combinations of epidermal growth factor receptor tyrosine kinase antagonists with conventional cytotoxic agents using median effect analysis. Anticancer Drugs 17: 921–928 16940802 10.1097/01.cad.0000224457.36522.60

[bib6] Ceresoli GL, Zucali PA, Favaretto AG, Grossi F, Bidoli P, Del Conte G, Ceribelli A, Bearz A, Morenghi E, Cavina R, Marangolo M, Parra HJ, Santoro A (2006) Phase II study of pemetrexed plus carboplatin in malignant pleural mesothelioma. J Clin Oncol 24: 1443–1448 16549838 10.1200/JCO.2005.04.3190

[bib7] Chu E, Koeller DM, Casey JL, Drake JC, Chabner BA, Elwood PC, Zinn S, Allegra CJ (1991) Autoregulation of human thymidylate synthase messenger RNA translation by thymidylate synthase. Proc Natl Acad Sci USA 88: 8977–8981 1924359 10.1073/pnas.88.20.8977 PMC52634

[bib8] Ciardiello F, Caputo R, Damiano V, Caputo R, Troiani T, Vitagliano D, Carlomagno F, Veneziani BM, Fontanini G, Bianco AR, Tortora G (2003) Antitumor effects of ZD6474, a small molecule vascular endothelial growth factor receptor tyrosine kinase inhibitor, with additional activity against epidermal growth factor receptor tyrosine kinase. Clin Cancer Res 9: 1546–1556 12684431

[bib9] de Boer RH, Arrieta Ó, Yang CH, Gottfried M, Chan V, Raats J, de Marinis F, Abratt RP, Wolf J, Blackhall FH, Langmuir P, Milenkova T, Read J, Vansteenkiste JF (2011) Vandetanib plus pemetrexed for the second-line treatment of advanced non-small-cell lung cancer: a randomized, double-blind phase III trial. J Clin Oncol 29: 1067–1074 21282537 10.1200/JCO.2010.29.5717

[bib10] Destro A, Ceresoli GL, Falleni M, Zucali PA, Morenghi E, Bianchi P, Pellegrini C, Cordani N, Vaira V, Alloisio M, Rizzi A, Bosari S, Roncalli M (2006) EGFR overexpression in malignant pleural mesothelioma. An immunohistochemical and molecular study with clinico-pathological correlations. Lung Cancer 51: 207–215 16384623 10.1016/j.lungcan.2005.10.016

[bib11] Dougherty U, Sehdev A, Cerda S, Mustafi R, Little N, Yuan W, Jagadeeswaran S, Chumsangsri A, Delgado J, Tretiakova M, Joseph L, Hart J, Cohen EE, Aluri L, Fichera A, Bissonnette M (2008) Epidermal growth factor receptor controls flat dysplastic aberrant crypt foci development and colon cancer progression in the rat azoxymethane model. Clin Cancer Res 14: 2253–2262 18413814 10.1158/1078-0432.CCR-07-4926

[bib12] Edwards JG, Swinson DE, Jones JL, Muller S, Waller DA, O’Byrne KJ (2003) Tumor necrosis correlates with angiogenesis and is a predictor of poor prognosis in malignant mesothelioma. Chest 124: 1916–1923 14605068 10.1378/chest.124.5.1916

[bib13] Feng FY, Varambally S, Tomlins SA, Chun PY, Lopez CA, Li X, Davis MA, Chinnaiyan AM, Lawrence TS, Nyati MK (2007) Role of epidermal growth factor receptor degradation in gemcitabine-mediated cytotoxicity. Oncogene 26: 3431–3439 17146438 10.1038/sj.onc.1210129

[bib14] Fujishima H, Nakano S, Masumoto N, Esaki T, Tatsumoto T, Kondo T, Niho Y (1997) Inhibition by 5-fluorouracil of ERCC1 and gamma-glutamylcysteine synthetase messenger RNA expression in a cisplatin-resistant HST-1 human squamous carcinoma cell line. Oncol Res 9: 167–172 9268987

[bib15] Gaafar R, Bahnassy A, Abdelsalam I, Kamel MM, Helal A, Abdel-Hamid A, Eldin NA, Mokhtar N (2010) Tissue and serum EGFR as prognostic factors in malignant pleural mesothelioma. Lung Cancer 70: 43–50 20347505 10.1016/j.lungcan.2010.01.002

[bib16] Garland LL, Rankin C, Gandara DR, Rivkin SE, Scott KM, Nagle RB, Klein-Szanto AJ, Testa JR, Altomare DA, Borden EC (2007) Phase II study of erlotinib in patients with malignant pleural mesothelioma: a Southwest Oncology Group Study. J Clin Oncol 25: 2406–2413 17557954 10.1200/JCO.2006.09.7634

[bib17] Giovannetti E, Lemos C, Tekle C, Smid K, Nannizzi S, Rodriguez JA, Ricciardi S, Danesi R, Giaccone G, Peters GJ (2008) Molecular mechanisms underlying the synergistic interaction of erlotinib, an epidermal growth factor receptor tyrosine kinase inhibitor, with the multitargeted antifolate pemetrexed in non-small-cell lung cancer cells. Mol Pharmacol 73: 1290–1300 18187583 10.1124/mol.107.042382

[bib18] Giovannetti E, Zucali PA, Peters GJ, Cortesi F, D’Incecco A, Smit EF, Falcone A, Burgers JA, Santoro A, Danesi R, Giaccone G, Tibaldi C (2010) Association of polymorphisms in AKT1 and EGFR with clinical outcome and toxicity in non-small cell lung cancer patients treated with gefitinib. Mol Cancer Ther 9: 581–593 20159991 10.1158/1535-7163.MCT-09-0665

[bib19] Govindan R, Kratzke RA, Herndon JE, Niehans GA, Vollmer R, Watson D, Green MR, Kindler HL, Cancer and Leukemia Group B (CALGB 30101) (2005) Gefitinib in patients with malignant mesothelioma: a phase II trial by the Cancer and Leukemia Group B. Clin Cancer Res 11: 2300–2304 15788680 10.1158/1078-0432.CCR-04-1940

[bib20] Guichard S, Hennebelle I, Bugat R, Canal P (1998) Cellular interactions of 5-fluorouracil and the camptothecin analogue CPT-11 (irinotecan) in a human colorectal carcinoma cell line. Biochem Pharmacol 55: 667–676 9515577 10.1016/s0006-2952(97)00541-8

[bib21] Gutierrez ME, Kummar S, Giaccone G (2009) Next generation oncology drug development: opportunities and challenges. Nat Rev Clin Oncol 6: 259–265 19390552 10.1038/nrclinonc.2009.38 PMC7079165

[bib22] Hanrahan EO, Lin HY, Kim ES, Yan S, Du DZ, McKee KS, Tran HT, Lee JJ, Ryan AJ, Langmuir P, Johnson BE, Heymach JV (2010) Distinct patterns of cytokine and angiogenic factor modulation and markers of benefit for vandetanib and/or chemotherapy in patients with non-small-cell lung cancer. J Clin Oncol 28: 193–201 19949019 10.1200/JCO.2009.22.4279 PMC3040010

[bib23] Heymach JV, Paz-Ares L, De Braud F, Sebastian M, Stewart DJ, Eberhardt WE, Ranade AA, Cohen G, Trigo JM, Sandler AB, Bonomi PD, Herbst RS, Krebs AD, Vasselli J, Johnson BE (2008) Randomized phase II study of vandetanib alone or with paclitaxel and carboplatin as first-line treatment for advanced non-small-cell lung cancer. J Clin Oncol 26: 5407–5415 18936474 10.1200/JCO.2008.17.3138

[bib24] Hodgson JT, McElvenny DM, Darnton AJ, Price MJ, Peto J (2005) The expected burden of mesothelioma mortality in Great Britain from 2002 to 2050. Br J Cancer 92: 587–593 15668716 10.1038/sj.bjc.6602307 PMC2362088

[bib25] Huang CL, Liu D, Nakano J, Yokomise H, Ueno M, Kadota K, Wada H (2007) E2F1 overexpression correlates with thymidylate synthase and survivin gene expressions and tumor proliferation in non-small-cell lung cancer. Clin Cancer Res 13: 6938–6946 18056168 10.1158/1078-0432.CCR-07-1539

[bib26] Janmaat ML, Rodriguez JA, Gallegos-Ruiz M, Kruyt FA, Giaccone G (2006) Enhanced cytotoxicity induced by gefitinib and specific inhibitors of the Ras or phosphatidyl inositol-3 kinase pathways in non-small cell lung cancer cells. Int J Cancer 118: 209–214 16003751 10.1002/ijc.21290

[bib27] Jimeno A, Kulesza P, Kincaid E, Bouaroud N, Chan A, Forastiere A, Brahmer J, Clark DP, Hidalgo M (2006) C-fos assessment as a marker of anti-epidermal growth factor receptor effect. Cancer Res 66: 2385–2390 16489045 10.1158/0008-5472.CAN-05-2882

[bib28] Karrison T, Kindler HL, Gandara DR, Lu C, Guterz TL, Nichols K, Chen H, Stadler WM, Vokes E (2007) Final analysis of a multi-center, double-blind, placebo-controlled, randomized phase II trial of gemcitabine/cisplatin plus bevacizumab or placebo in patients (pts) with malignant mesothelioma (abstract). Proc Am Soc Clin Oncol 25(18S): 7526

[bib29] Kindler HL (2008) Systemic treatments for mesothelioma: standard and novel. Curr Treat Options Oncol 9: 171–179 18770046 10.1007/s11864-008-0071-3 PMC2782121

[bib30] Kobayashi S, Shimamura T, Monti S, Steidl U, Hetherington CJ, Lowell AM, Golub T, Meyerson M, Tenen DG, Shapiro GI, Halmos B (2006) Transcriptional profiling identifies cyclin D1 as a critical downstream effector of mutant epidermal growth factor receptor signaling. Cancer Res 66: 11389–11398 17145885 10.1158/0008-5472.CAN-06-2318

[bib31] Li T, Ling YH, Goldman ID, Perez-Soler R (2007) Schedule-dependent cytotoxic synergism of pemetrexed and erlotinib in human non-small cell lung cancer cells. Clin Cancer Res 13: 3413–3422 17545550 10.1158/1078-0432.CCR-06-2923

[bib32] Linder C, Linder S, Munck-Wikland E, Strander H (1998) Independent expression of serum vascular endothelial growth factor (VEGF) and basic fibroblast growth factor (bFGF) in patients with carcinoma and sarcoma. Anticancer Res 18: 2063–2068 9677468

[bib33] Lo HW, Hung MC (2006) Nuclear EGFR signalling network in cancers: linking EGFR pathway to cell cycle progression, nitric oxide pathway and patient survival. Br J Cancer 94: 184–188 16434982 10.1038/sj.bjc.6602941 PMC2361115

[bib34] Magné N, Fischel JL, Dubreuil A, Formento P, Ciccolini J, Formento JL, Tiffon C, Renée N, Marchetti S, Etienne MC, Milano G (2003) ZD1839 (Iressa) modifies the activity of key enzymes linked to fluoropyrimidine activity: rational basis for a new combination therapy with capecitabine. Clin Cancer Res 9: 4735–4742 14581344

[bib35] Matsumoto S, Igishi T, Hashimoto K, Kodani M, Shigeoka Y, Nakanishi H, Touge H, Kurai J, Makino H, Takeda K, Yasuda K, Hitsuda Y, Shimizu E (2004) Schedule-dependent synergism of vinorelbine and 5-fluorouracil/UFT against non-small cell lung cancer. Int J Oncol 25: 1311–1318 15492820

[bib36] Morelli MP, Cascone T, Troiani T, De Vita F, Orditura M, Laus G, Eckhardt SG, Pepe S, Tortora G, Ciardiello F (2005) Sequence-dependent antiproliferative effects of cytotoxic drugs and epidermal growth factor receptor inhibitors. Ann Oncol 16(Suppl 4): 61–68 10.1093/annonc/mdi910 15923432

[bib37] Nannizzi S, Veal GJ, Giovannetti E, Mey V, Ricciardi S, Ottley CJ, Del Tacca M, Danesi R (2010) Cellular and molecular mechanisms for the synergistic cytotoxicity elicited by oxaliplatin and pemetrexed in colon cancer cell lines. Cancer Chemother Pharmacol 66: 547–558 20020129 10.1007/s00280-009-1195-2 PMC2886085

[bib38] Novakovic P, Stempak JM, Sohn KJ, Kim YI (2006) Effects of folate deficiency on gene expression in the apoptosis and cancer pathways in colon cancer cells. Carcinogenesis 27: 916–924 16361273 10.1093/carcin/bgi312

[bib39] Nutt JE, O’Toole K, Gonzalez D, Lunec J (2009) Growth inhibition by tyrosine kinase inhibitors in mesothelioma cell lines. Eur J Cancer 45: 1684–1691 19318229 10.1016/j.ejca.2009.02.022

[bib40] Nutt JE, Razak AR, O’Toole K, Black F, Quinn AE, Calvert AH, Plummer ER, Lunec J (2010) The role of folate receptor alpha (FRalpha) in the response of malignant pleural mesothelioma to pemetrexed-containing chemotherapy. Br J Cancer 102: 553–560 20051956 10.1038/sj.bjc.6605501 PMC2822938

[bib41] Ogino H, Yano S, Kakiuchi S, Yamada T, Ikuta K, Nakataki E, Goto H, Hanibuchi M, Nishioka Y, Ryan A, Sone S (2008) Novel dual targeting strategy with vandetanib induces tumor cell apoptosis and inhibits angiogenesis in malignant pleural mesothelioma cells expressing RET oncogenic rearrangement. Cancer Lett 265: 55–66 18364248 10.1016/j.canlet.2008.02.018

[bib42] Peters GJ, Backus HH, Freemantle S, van Triest B, Codacci-Pisanelli G, van der Wilt CL, Smid K, Lunec J, Calvert AH, Marsh S, McLeod HL, Bloemena E, Meijer S, Jansen G, van Groeningen CJ, Pinedo HM (2002) Induction of thymidylate synthase as a 5-fluorouracil resistance mechanism. Biochim Biophys Acta 1587: 194–205 12084461 10.1016/s0925-4439(02)00082-0

[bib43] Rabo YB, Shoshan MC, Linder S, Hansson J (1996) Different mechanisms are responsible for c-jun mRNA induction by cisplatin and ultraviolet light. Int J Cancer 65: 821–826 8631598 10.1002/(SICI)1097-0215(19960315)65:6<821::AID-IJC19>3.0.CO;2-6

[bib44] Righi L, Papotti MG, Ceppi P, Billè A, Bacillo E, Molinaro L, Ruffini E, Scagliotti GV, Selvaggi G (2010) Thymidylate synthase but not excision repair cross-complementation group 1 tumor expression predicts outcome in patients with malignant pleural mesothelioma treated with pemetrexed-based chemotherapy. J Clin Oncol 28: 1534–1539 20177021 10.1200/JCO.2009.25.9275

[bib45] Shuai XM, Han GX, Wang GB, Chen JH (2006) Cyclin D1 antisense oligodexoyneucleotides inhibits growth and enhances chemosensitivity in gastric carcinoma cells. World J Gastroenterol 12: 1766–1769 16586549 10.3748/wjg.v12.i11.1766 PMC4124355

[bib46] Stoppoloni D, Canino C, Cardillo I, Verdina A, Baldi A, Sacchi A, Galati R (2010) Synergistic effect of gefitinib and rofecoxib in mesothelioma cells. Mol Cancer 9: 27 20122271 10.1186/1476-4598-9-27 PMC2828989

[bib47] Suenaga M, Yamaguchi A, Soda H, Orihara K, Tokito Y, Sakaki Y, Umehara M, Terashi K, Kawamata N, Oka M, Kohno S, Tei C(2006) Antiproliferative effects of gefitinib are associated with suppression of E2F-1 expression and telomerase activity. Anticancer Res 26: 3387–3391 17094457

[bib48] Tekle C, Giovannetti E, Sigmond J, Graff JR, Smid K, Peters GJ (2008) Molecular pathways involved in the synergistic interaction of the PKC beta inhibitor enzastaurin with the antifolate pemetrexed in non-small cell lung cancer cells. Br J Cancer 99: 750–759 18728666 10.1038/sj.bjc.6604566 PMC2528136

[bib49] Van Schaeybroeck S, Karaiskou-McCaul A, Kelly D, Longley D, Galligan L, Van Cutsem E, Johnston P (2005) Epidermal growth factor receptor activity determines response of colorectal cancer cells to gefitinib alone and in combination with chemotherapy. Clin Cancer Res 11: 7480–7489 16243822 10.1158/1078-0432.CCR-05-0328

[bib50] Van Schaeybroeck S, Kyula J, Kelly DM, Karaiskou-McCaul A, Stokesberry SA, Van Cutsem E, Longley DB, Johnston PG (2006) Chemotherapy-induced epidermal growth factor receptor activation determines response to combined gefitinib/chemotherapy treatment in non-small cell lung cancer cells. Mol Cancer Ther 5: 1154–1165 16731747 10.1158/1535-7163.MCT-05-0446

[bib51] Vogelzang NJ, Rusthoven JJ, Symanowski J, Denham C, Kaukel E, Ruffie P, Gatzemeier U, Boyer M, Emri S, Manegold C, Niyikiza C, Paoletti P (2003) Phase III study of pemetrexed in combination with cisplatin versus cisplatin alone in patients with malignant pleural mesothelioma. J Clin Oncol 21: 2636–2644 12860938 10.1200/JCO.2003.11.136

[bib52] Yokoi K, Sasaki T, Bucana CD, Fan D, Baker CH, Kitadai Y, Kuwai T, Abbruzzese JL, Fidler IJ (2005) Simultaneous inhibition of EGFR, VEGFR, and platelet-derived growth factor receptor signaling combined with gemcitabine produces therapy of human pancreatic carcinoma and prolongs survival in an orthotopic nude mouse model. Cancer Res 65: 10371–10380 16288027 10.1158/0008-5472.CAN-05-1698 PMC1456803

[bib53] Zucali PA, Ceresoli GL, De Vincenzo F, Simonelli M, Lorenzi E, Gianoncelli L, Santoro A (2011a) Advances in the biology of malignant pleural mesothelioma. Cancer Treat Rev 37: 543–558 21288646 10.1016/j.ctrv.2011.01.001

[bib54] Zucali PA, Giovannetti E, Destro A, Mencoboni M, Ceresoli GL, Gianoncelli L, Lorenzi E, De Vincenzo F, Simonelli M, Perrino M, Bruzzone A, Thunnissen E, Tunesi G, Giordano L, Roncalli M, Peters GJ, Santoro A (2011b) Thymidylate synthase and excision repair-cross-complementing group-1 as predictors of responsiveness in mesothelioma patients treated with pemetrexed-carboplatin. Clin Cancer Res 17: 2581–2590 21262916 10.1158/1078-0432.CCR-10-2873

